# Effect of Methylphenidate on State Anxiety in Children With ADHD-A Single Dose, Placebo Controlled, Crossover Study

**DOI:** 10.3389/fnbeh.2019.00106

**Published:** 2019-05-15

**Authors:** Maya Kritchman, May Koubi, Aviva Mimouni Bloch, Yuval Bloch

**Affiliations:** ^1^The Emotion-Cognition Research Center, Shalvata Mental Health Care Center, Hod-Hasharon, Israel; ^2^Sackler Faculty of Medicine, Tel-Aviv University, Tel-Aviv, Israel; ^3^Child and Adolescent Outpatient Clinic, Shalvata Mental Health Care Center, Hod-Hasharon, Israel; ^4^The Pediatric Neurology and Developmental Unit, Loewenstein Rehabilitation Hospital, Raanana, Israel

**Keywords:** ADHD, state anxiety, methylphenidate, attention-deficit hyperactivity disorder, pharmacotherapy

## Abstract

**Introduction:** Non-adherence to efficacious pharmacotherapy is a major obstacle in the treatment of children suffering from attention deficit hyperactive disorder (ADHD). Some hold the position that pharmacotherapy induces anxiety, and that this is one of the reasons for this non-adherence. Previous studies have pointed to the opposite, a moderating effect of methylphenidate (MPH) on state anxiety in patients with ADHD. This has been shown in continuous treatment in children, but not on a single dose. We hypothesized that a single dose might have a different effect.

**Method:** Twenty children with ADHD were given single doses of MPH in a randomized, controlled, crossover, double blind study. State anxiety using The Spielberger State-Trait Anxiety Inventory (STAI) and a continuous performance test were assessed.

**Results:** As a group, no change was detected in state anxiety with MPH or placebo. However, children who were given MPH during the first session as opposed to those who received placebo first, demonstrated deterioration in baseline state anxiety in the second session [*t*_(2.485)_, *p* < 0.05].

**Conclusion:** Our findings show a possible delayed anxiety-provoking effect of a single dose of MPH. This may be relevant to the understanding of difficulties in adherence with MPH treatment in children with ADHD.

**Clinical Trial Registration:** www.ClinicalTrials.gov, identifier: NCT01798459

## Introduction

Attention deficit hyperactive disorder (ADHD) is a common worldwide behavioral and neurocognitive disorder with an onset in childhood and substantial negative effects on child and adult life (Erskine et al., 2016; Sayal et al., 2018). The evidence regarding the efficacy and importance of pharmacotherapy in the treatment of ADHD is extremely robust (Group, 1999; Lichtenstein et al., 2012; Chang et al., 2017), and pharmacotherapy (most commonly prescribed are stimulants) is part of treatment recommendations and practice guidelines (Atkinson and Hollis, 2010; Subcommittee on Attention-Deficit/Hyperactivity Disorder et al., 2011; Seixas et al., 2012).

Studies demonstrate high rates of ADHD comorbidity with anxiety (~25–50%) (Larson et al., 2011; Sciberras et al., 2014). The effect of stimulant medications on anxiety in ADHD patients, bears both conceptual and clinical importance. Non-adherence to pharmacotherapy is a major obstacle to effective treatment for children suffering from ADHD. Side effects that include loss of appetite, as well as “not feeling like myself” and feeling tense and stressed were identified as leading reasons for non-adherence (Frank et al., 2015; Kovshoff et al., 2016; Wang et al., 2016). MPH causes a rise in extracellular dopamine in the brain, leading to improvements in cognitive functions such as attention and vigilance. However, it may possibly also cause a rise in tension, stress and anxiety through the same mechanism. Evidence regarding the possibility of anxiety as a stimulant side effect have been mixed. There are patient reports that highlight that anxiety is a possible side effect of stimulants, however, systematic studies point to the opposite effect. A meta-analysis of well controlled studies that collected systematic reports of anxiety during stimulant therapy revealed a significant dose-dependent reduction in anxiety in the treatment group when compared with placebo (Coughlin et al., 2015). This raises the possibility that untreated ADHD is a mediator of anxiety.

One possible explanation for these contradictory findings is the difference in duration of follow-up. In a nationwide follow-up study of adherence to stimulants in Taiwan, more than 17% of the children that were diagnosed with ADHD and given a prescription for Methylphenidate IR (MPH), discontinued therapy after a single dose, accounting for approximately a third of the total discontinuation rate within the first year (51% of study population discontinued within the first year) (Wang et al., 2016). Thus, it is possible that there is an acute rise in anxiety levels in children treated with MPH, however, for a considerable part of the patient population, continuous therapy can improve the anxiety related to ADHD.

In the present pilot study, we set out to assess state anxiety in pediatric ADHD patients facing a cognitive task, after being given single dose of MPH, compared with placebo.

## Methodology

### Participants

A group of 20 children and adolescents native Hebrew speakers, 11 boys and 9 girls, between ages 8 and 18 years, participated in the study (M age = 10.5 years, SD = 1.99). The patients were recruited through advertisement and through the outpatient clinic. The diagnosis of ADHD was based on structured clinical interviews with the child and parents conducted by a child psychiatrist trained in diagnosing childhood ADHD with the aid of the Swanson, Nolan, and Pelham Rating Scale (SNAP-IV) filled by multiple informants. This questionnaire is widely used, as a screening tool, with good sensitivity to detect the core symptoms of attention deficit hyperactivity disorder (ADHD) (Hall et al., 2019). Criteria for exclusion were a known diagnosis of autistic spectrum disorder, schizophrenia, bipolar disorder, current depressive episode, eating disorder, active anxiety disorder, and current (past 6 months) substance abuse. No formal cognitive assessment was performed, but all participants studied in regular classes, and based on the clinical assessment, there was no intellectual disability.

### Procedure

This was a randomized, double-blind, placebo-controlled crossover trial. Study protocol and consent form were approved by both the institutional review board (0009-12-SHA) and the national review board (20120239). Participants were recruited from our ADHD clinic. The study protocol was explained in detail to the participant and family. Informed consent was provided by participants' parent or legal guardian prior to any study-related activity.

The study was registered on clinical trials.gov 0009-12-SHA; NCT01798459.

All participants underwent two identical session evaluations. In both sessions, the subjects completed The Spielberger State-Trait Anxiety Inventory (STAI) questionnaire (Okun et al., 1996) before any intervention and performed Cambridge Neuropsychological Test Automated Battery CANTAB neurocognitive tests (De Luca et al., 2003). Mean STAI-Trait was 45.90 (SD = 6.70).

Participants were randomly assigned to receive MPH 0.3 mg/kg immediate release or inert ingredient (placebo). Forty-five minutes after drug administration, subjects completed STAI—state questionnaire again and performed the cognitive tasks. In the second session, medication (MPH/placebo) was crossed-over.

### Tools

*The Swanson, Nolan, and Pelham questionnaire (SNAP-IV)* (Bussing et al., 2008), is a well validated questionnaire that is used to quantify ADHD symptomatology as well as to screen for other common behavioral and emotional symptoms. It was completed by one of the parents and, separately, by a teacher.

*The Mini International Neuropsychiatric Interview for Children and Adolescents* (Sheehan et al., 2010), is a short structured diagnostic interview for phenomenological psychiatric disorders in children and adolescents.

*The Spielberger State-Trait Anxiety Inventory (STAI)* (Spielberger et al., 1970), is a 40-item scale which measures the intensity of felt anxiety. It includes 20 questions aimed to quantify state anxiety (temporary, experienced in particular situations) and 20 questions to evaluate trait anxiety (a general tendency to perceive situations as threatening). Each item is rated on a 1–4 scale. Range of scores for each subtest is 20–80, the higher score indicating greater anxiety (Oei et al., 1990; Rossi and Pourtois, 2012). The STAI was used in its' hebrew version (Teichman and Melink, 1984), that was validated and used in previous studies in this age group (Zohar and Bruno, 1997).

The following Subsets from *Cambridge Neuropsychological Test Automated Battery (CANTAB)* were administered: *The Motor screening Test (MOT), The Rapid Visual Information Processing (RVP), The Spatial Working Memory (SWM), and the Reaction Time (RT) tasks*. These tasks were chosen because they measure executive functions relevant in evaluating ADHD and commonly used both in research and in clinical practice of ADHD. The reason for their use was the thought that they will be challenging and thus anxiety provoking for patients suffering from ADHD.

### Statistical Analysis

A paired *t*-test was applied for within subject comparison. Based on previous studies in adults we expected a sample size of 20 participants to suffice (Bloch et al., 2017; Pozzi et al., 2018).

## Results

No difference was found in state anxiety after administration of MPH (before 44.15 (±5.24) after 45.15 (±4.77) [*t*_(−0.925)_, *p* = 0.367]; nor following administration of placebo (before 46.25 (±5.40) after 45.95 (±4.12) [*t*_(1.03)_, *p* = 0.317].

However, baseline state anxiety scores at the second visit was significantly associated with previous experience at the first visit. Those who received MPH at the first visit (baseline anxiety 44 (±5/12) reported higher baseline state anxiety at the second visit (46.22 ± (4.79) before administration of the placebo) [*t*_(2.485)_, *p* < 0.05).

The difference in baseline state anxiety scores for the participants who were given placebo in the first session was a non-significant decline [*t*_(1.724)_, *p*= 0.115].

## Discussion

In the present study, there was no immediate effect of a single dose of MPH on state anxiety in ADHD pediatric patients. However, there was an incline in baseline anxiety at the second treatment visit in patients that received MPH at the first visit.

This may be considered to be a type of conditioned phenomenon, related to the experience with the medication, whose importance is in the possible clinical relevance. Seventeen percent of the pediatric patients that start MPH, stop after the first prescription (Wang et al., 2016), raising the probability of early side effects as a reason for discontinuation of MPH.

Non-adherence to efficient pharmacotherapy is a major obstacle in treating ADHD patients. The reasons for non-adherence vary, but a major reason is side effects. While anxiety is suggested as an important side effect (Pozzi et al., 2018), studies and meta-analyses support the opposite, i.e., that prolonged MPH treatment for ADHD causes a reduction in anxiety (Coughlin et al., 2015; Pozzi et al., 2018).

Since most studies focused on prolonged therapy, they could not reveal an immediate anxiety provoking effect that is relevant to early medication withdrawal.

In a previous study of adult ADHD patients, our group demonstrated an anxiolytic effect of a single dose of MPH (Bloch et al., 2017). While one could argue that this relates to the age difference, it is important to stress that adult ADHD patients have, like the younger age groups, difficulties with adherence, and suffer from MPH-related side effects (Lichtenstein et al., 2012; Fredriksen and Peleikis, 2016). Thus, the age difference seems to not be supported as clinically-relevant to the different effect on anxiety. It is important to note that it was not an immediate anxiety provoking effect that we demonstrated in the current study.

The finding of this pilot study relates to a delayed possibly conditioned anxiety provoking effect, that has not been studied yet. It is different from the anxiolytic effect of MPH on adherent patients that probably do not suffer from the aversive effect we encounter with some pediatric ADHD patients in the clinic (Kovshoff et al., 2016; Pozzi et al., 2018). If supported by larger studies, this type of anxiety can contribute to our understanding of non-adherence, and help develop new approaches to overcome these difficulties.

There are several limitations of the current study. First and most importantly, the sample was small, and with considerable age variation. Larger and more age homogenous studies with imaging evaluations, are needed in order to substantiate these findings and have a wider understanding of their implications.

## Ethics Statement

The study was approved by the Shalvata ethics committee. Parents completed the informed consent process and signed the informed consent form. Children and adolescents signed the assent form.

## Author Contributions

All authors have made substantial contributions to the conception or design of the work. MKr and MKo made major contribution to the acquisition of data. All authors contributed to the analysis and interpretation of data for the work, drafting the work or revising it critically for important intellectual content, provide approval for publication of the content, and agree to be accountable for all aspects of the work in ensuring that questions related to the accuracy or integrity of any part of the work are appropriately investigated and resolved.

### Conflict of Interest Statement

The authors declare that the research was conducted in the absence of any commercial or financial relationships that could be construed as a potential conflict of interest.
